# Efficient Reduction in Methylene Blue Using Palladium Nanoparticles Supported by Melamine-Based Polymer

**DOI:** 10.3390/ma16175887

**Published:** 2023-08-28

**Authors:** Nazeeha S. Alkayal, Manal Ibrahim, Nada Tashkandi, Maha M. Alotaibi

**Affiliations:** Chemistry Department, Faculty of Science, King Abdulaziz University, P.O. Box 80203, Jeddah 21589, Saudi Arabia; maliibrahim@stu.kau.edu.sa (M.I.); nytashkandi@kau.edu.sa (N.T.); mralotaibi@kacst.edu.sa (M.M.A.)

**Keywords:** porous organic polymer, palladium nanoparticles, catalytic reduction, heterogeneous catalysis, methylene blue, wastewater treatment

## Abstract

In this work, palladium nanoparticles, supported by polyaminals (Pd@PAN-NA), were synthesized via a reverse double solvent approach and used as a nano catalyst. The thermogravimetric and the elemental analysis revealed that the catalyst had good dispersity and improved thermal stability. The catalytic activity of the prepared Pd@PAN-NA catalyst was studied for a methylene blue chemical reaction in the presence of NaBH_4_ as a reducing agent. The effect of the catalyst dose, pH, and dye initial concentration were examined to optimize the chemical reduction conditions. The prepared catalyst Pd@PAN-NA removed 99.8% of methylene blue organic dye, indicating its potential effect for treating waste and contaminated water.

## 1. Introduction

Dyes are one of the most environmentally polluting materials that result from industrial development in the fields of textiles, pharmaceuticals, cosmetics, printing, etc. Water polluted with dyes is discharged directly into the aquatic environment, which leads to numerous damages to the ecosystem, and thus, to the health of the human body. Methylene blue (MB) is a heterocyclic aromatic cationic dye that contains a thiazine group in its chemical structure. MB is among the most frequently used organic dyes and causes a variety of health hazards such as digestive, skin, and mental issues and other health problems. Therefore, practical solutions and technologies must be investigated to treat the water from the colored effluents [1,2,3,4,5,6,7].

There are different methods and technologies that have been routinely used for dye-bearing water treatment such as adsorption, membrane filtration, electrochemical treatment, photodegradation, and chemical coagulation. However, there are certain drawbacks to these therapeutic approaches; some of them suffer from inefficiency and cannot completely degrade the organic dyes. In addition, they lead to the generation of secondary pollution [8,9,10,11,12,13]. Chemical reduction using nanoparticle-based materials in the presence of a reducing agent such as NaBH_4_ is a preferable approach for the degradation of the dyes and other organic pollutants because of the facile and ease of operation, high degradation rate, complete removal of contaminants, and useful byproducts that can be utilized in different industries [14,15,16].

Nanoparticles are attractive materials because they possess extremely large surface-to-volume proportions, abundant active atoms at the surface, and high electron storage capacity [14,17]. However, due to their high surface energy, the nanoparticles tend to agglomerate to reach stability. Although the agglomeration phenomenon of the nanoparticles decreases their surface energy, the surface area reduces as well, which leads to a decrease in their activities toward different applications [18,19,20]. To solve these issues, suitable supports are used to stabilize the metal nanoparticles during synthesis and prevent agglomeration. Common examples of support materials that are used for this purpose include porous carbon, zeolites, metal−organic frameworks (MOFs), covalent organic frameworks (COFs), and porous organic polymers (POPs) [21,22]. Due to their structural stability, low density, and tunable porosity [23,24], POPs are regarded as excellent candidates for the metal nanoparticles’ immobilization. In addition, POPs are constructed from functionalized building blocks enriched with heteroatoms such as nitrogen, sulfur, and oxygen. Thus, POPs can immobilize and stabilize the metal nanoparticles within the internal and external pores through coordination ability. These heteroatoms inside the pores also guide the nucleation and growth of nanoparticles, which is beneficial for size and dispersity control [25,26,27,28,29].

Recently, as an alternative strategy to encapsulate the metal nanoparticles in POPs, a reverse double solvents approach (RDSA) was proposed [27,30]. The principle of this method is to use a small amount of hydrophobic solvent to incorporate the metal precursor inside the hydrophobic pores via diffusion in a large amount of hydrophilic solvent. Then, a high-concentration reducing agent is used to reduce the metal precursor to generate stable and dispersed metal nanoparticles [23,31,32]. Jin Yang and his group have fabricated an ultrafine and highly dispersed Pd@TP-POP catalyst using a reverse double solvent approach. The catalyst exhibited an outstanding performance for p-nitrophenol and aldehydes catalytic hydrogenation [33]. Man Yuan and co-workers reported PC-POPs with abundant triazinyl groups for supporting the Pd NPs with superior catalytic activities and which displayed no apparent agglomeration or leaching after 10 catalytic cycles [34]. However, reports on the stabilization of the metal nanoparticles on POP materials via the RDSA method and the study of their properties for heterogeneous catalysis applications are still rare. To the best of our knowledge, no report has been published on the use of the RDSA method to add metal nanoparticles onto melamine-based polyaminals.

Herein, polyaminal-based porous organic polymer (PAN-NA) was used as a support medium to stabilize the Pd nanoparticles (Pd@PAN-NA). The Pd@PAN-NA catalyst was prepared via the RDSA for the first time and analyzed with powder X-ray diffraction (PXRD), thermogravimetry (TGA), Scanning electron microscopy (SEM), The transmission electron microscopy (TEM), and N_2_ adsorption–desorption methods. The catalyst was applied for methylene blue reduction from wastewater. Different variables like pH, catalyst dose, and dye initial concentrations were studied to optimize the reaction conditions.

## 2. Method

### 2.1. Materials

Palladium acetate Pd(OAc)_2_ (99.9%) was purchased from Sigma-Aldrich, dichloromethane (CH_2_Cl_2_) (~99.9%) was purchased from Fisher chemicals, NaOH (98%) and NaBH_4_ (99%) were supplied by BDH chemicals, HCl (35%) was supplied from LOBA Chemie, and methylene blue (98%) was provided by Laboratory Reagent. All substances were used without additional purification.

### 2.2. Synthesis of PAN-NA

PAN-NA polymer was prepared according to the method in our previous research [35].

### 2.3. Synthesis of Pd@PAN-NA Catalyst by RDSA

Typically, 85 mg of PAN-NA powder was weighed and dispersed in 20 mL of deionized water and vigorously stirred for 30 min. After that, a hydrophobic solution of Pd(Oac)_2_/CH_2_Cl_2_ (0.02 mL) with a Pd(Oac)_2_ content of (0.01 mmol) was added drop by drop into the dispersion, and the prepared mixture was sonicated for 3 h. Finally, a high concentration of a fresh NaBH_4_/H_2_O solution (0.5 mL/2.6 M) was added rapidly to reduce the Pd^2+^ to Pd^0^ and stirred for 3 h. The prepared catalyst was filtered and washed with deionized water. Then, the solid was left to dry in the air for 24 h and further used in the MB catalytic reduction.

### 2.4. Characterization

Scanning electron microscopy imaging and energy dispersive X-ray spectroscopy (EDX) were performed with a FEI TENEO VS microscope equipped with an EDAX detector. The polymer sample was mounted on the aluminum stub using adhesive carbon tape and sputter coated with 3 nm of iridium to avoid sample charging during imaging. Powder-X-ray diffraction patterns were studied using a Bruker D8 Advance with Cu Ka radiation (wavelength 1.5418 A) at 40 kV and 40 mA. The patterns were collected between 2θ of 10° and 80°, and the scan speed was 1.5/min. The N_2_ adsorption–desorption measurement was conducted on a Micromeritics 3Flex 3500. The Brunauer–Emmett–Teller (BET) and Langmuir methods were employed to calculate the surface area of the material. The t-plot is used to approximate the micropore surface area. Before analysis, the sample was degassed via heating to 120 °C for 12 h under vacuum. The nonlocal density functional theory (NLDFT) was utilized to determine the pore size distribution (PSD). The UV-vis spectra were recorded using a spectrophotometer UV-1501.

### 2.5. Methylene Blue Reduction

3 mL of the 0.1 mM MB solution was mixed with 3 mg of NaBH_4_ in a quartz cuvette. Then, 0.05 mL of the 2 mg/mL dispersed catalyst (~30 min in the sonicator) was injected into the solution. The cuvette was placed in the UV-vis spectrophotometer and the reduction reaction was monitored. The MB absorbance was measured every minute until the characteristic peak of the dye disappeared. The effect of the different parameters (catalyst dose, pH, dye concentration) was studied to recognize the optimum reduction reaction conditions. All the solutions and dispersions were prepared in deionized water.

The degradation efficiency (D%) was measured using the following equation:

$$D\%=\frac{A_{0}-A_{t}}{A_{0}}\times 100$$

where A_0_ and A_t_ refer to the absorbances at time 0 and t, respectively. The reduction in MB with NaBH_4_ was examined in the absence and existence of the Pd@PAN-NA catalyst. To further confirm the role of Pd nanoparticles for the effective reduction in MB, the supported polymer (PAN-NA) was tested in the presence of NaBH_4_.

#### 2.5.1. Effect of Catalyst Dose

To study the influence of the catalyst dose on increasing the reduction rate of MB, the experiments were performed under the following reaction conditions: methylene blue concentration of 0.1 mM, NaBH_4_ mass of 3 mg, and catalyst amount of 0.05 mL with different concentrations (mg/mL) of 0.9, 1.5, 2 (initial), and 3.5, respectively.

#### 2.5.2. Effect of pH

The medium pH is another significant factor that affects the chemical reduction in MB. The pH values of 5, 6.6 (initial), 7.9, 9.2, and 10.9 was adjusted by 0.1 M of the NaOH and HCl solutions, and the other parameters were kept unchanged (initial dye concentration of 0.1 mM, NaBH_4_ mass of 3 mg, catalyst dose of 3.5 mg/mL, and time of 6 min).

#### 2.5.3. Effect of Dye Concentration

To evaluate the influence of the initial dye concentration on the catalytic reduction in MB, different solutions of MB were prepared with concentrations (mM) of 0.02, 0.08, 0.1 (initial), 0.2, and 0.3. The experiments were applied at the optimum values of the previous parameters.

## 3. Results and Discussion

### 3.1. Synthesis and Characterization of Pd@PAN-NA Catalyst

**Scheme 1:**
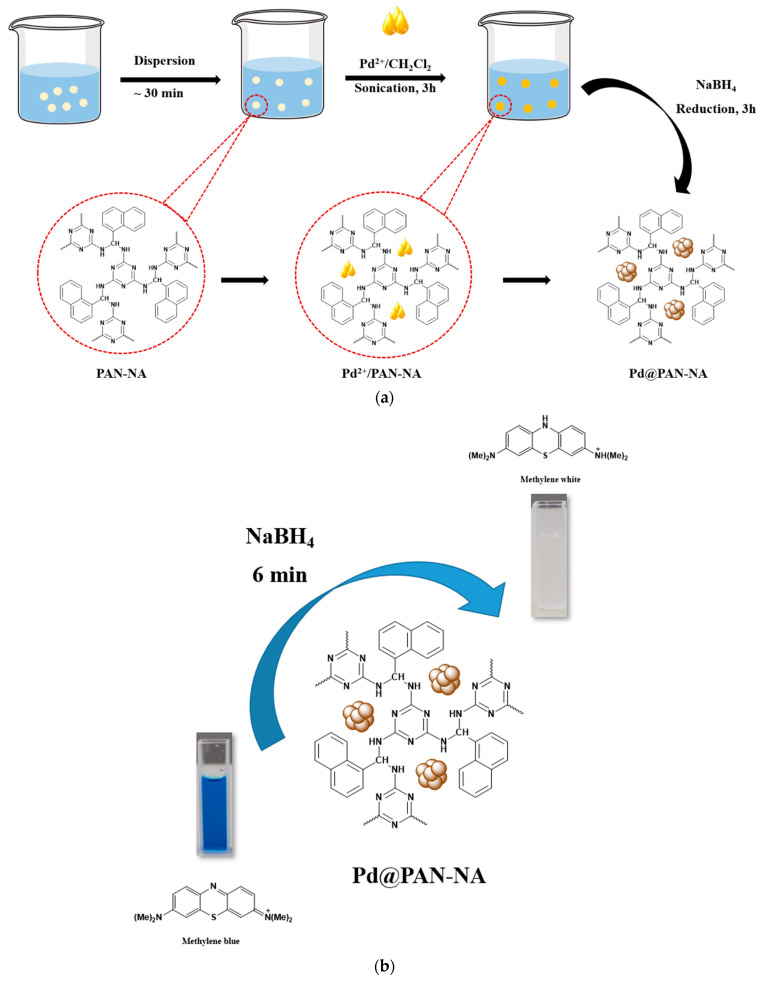
(**a**) Schematic synthetic procedure of Pd@PAN-NA via the RDSA method, and (**b**) the catalytic reduction in MB.

In our previous work, the polyaminal-based porous organic polymer (PAN-NA) was synthesized from melamine and naphthaldehyde monomers via a one-pot polycondensation reaction [35]. The PAN-NA polymer was characterized by different techniques including Fourier transform infrared spectroscopy, solid-state ^13^C NMR, powder X-ray diffraction, thermogravimetry, field-emission scanning electron microscope, and N_2_ adsorption–desorption methods. The results exhibited the successful fabrication of PAN-NA with a large surface area of 607.46 m^2^/g and inherent microporosity (0.54–1.27 nm). The PAN-NA polymer was used as an efficient adsorbent for the metal uptake from wastewater. The nitrogen atoms in triazine units and aminal linkage acts as binding sites to interact with the metal ions. Based on our previous result, it is expected that the PAN-NA polymer is excellent support for uploading the metal NPs. Thus, the capability of the immobilization of the palladium nanoparticles onto the polymer was tested. Firstly, the catalyst was synthesized via the RDSA method as follows: The PAN-NA polymer was dispersed in deionized water, then the Pd(OAc)_2_/CH_2_Cl_2_ (2.6 M) solution was added to the dispersion and the mixture was placed in a sonicator. After 3 h, the palladium ions were reduced to atoms via the NaBH_4_ reducing agent to finally produce the gray powder Pd@PAN-NA catalyst (Scheme 1). To confirm the successful construction of the catalyst, Pd@PAN-NA was characterized by PXRD, SEM, TEM, EDX, TGA, and N_2_ adsorption–desorption methods.

**Figure 1:**
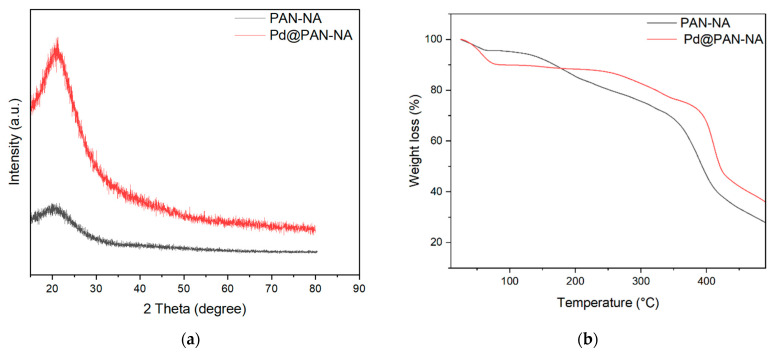
(**a**) PXRD patterns and (**b**) TGA curves of PAN-NA and Pd@PAN-NA.

**Figure 2:**
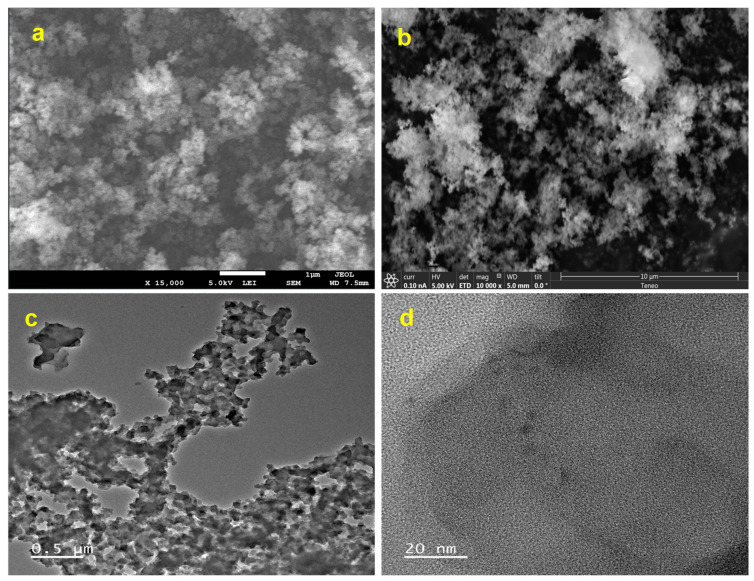
SEM images of (**a**) PAN-NA and (**b**) Pd@PAN-NA. TEM images with different magnifications of (**c**,**d**) Pd@PAN-NA.

The PXRD patterns in Figure 1a show a broad peak attributed to the amorphous PAN-NA that does not change in the Pd@PAN-NA catalyst, which confirms its stability and suitability to be used as support. No peaks referring to the Pd nanoparticles were observed. The thermal stability of Pd@PAN-NA was tested using TGA (Figure 1b), and after the incorporation of Pd NPs, the stability of the polymer was improved. The 10% weight loss at 74.1 °C for Pd@PAN-NA is primarily ascribed to the removal of the residue of water and CH_2_Cl_2_ solvents. The major degradation of Pd@PAN-NA occurs around 400 °C. This result was indicative of the high thermal stability of the Pd@PAN-NA catalyst. The Pd@PAN-NA morphology was investigated using SEM analysis. The result demonstrates that the PAN-NA support has a cotton-like shape with an irregular tiny particles structure, and these morphologies are preserved after the introduction of the Pd nanoparticles (Figure 2a,b). The TEM images (Figure 2c) indicate that the Pd nanoparticles were successively immobilized on the surface of the polymer support with good dispersity and an average particle size of ~57 nm. Also, a few small Pd NPs were observed with an average particle size of 3.5 nm, as illustrated in Figure 2d.

**Figure 3:**
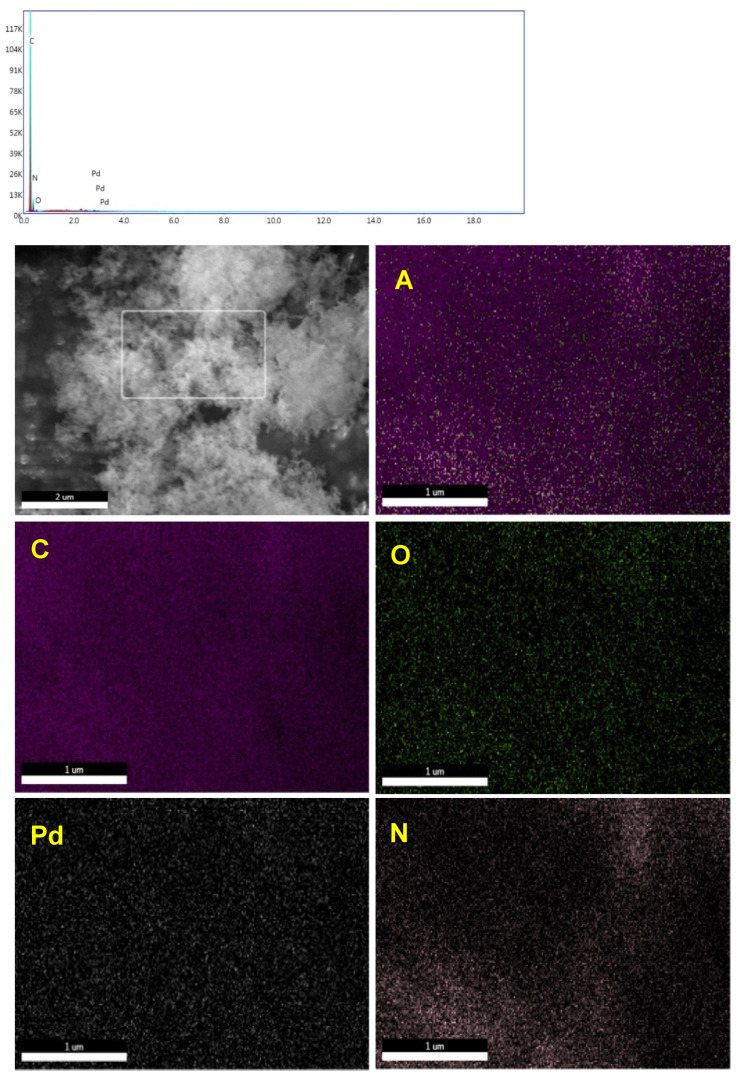
EDX elemental mapping of (A) a combination of all elements, C mapping image, O mapping image, Pd mapping image, and N mapping image, respectively.

**Figure 4:**
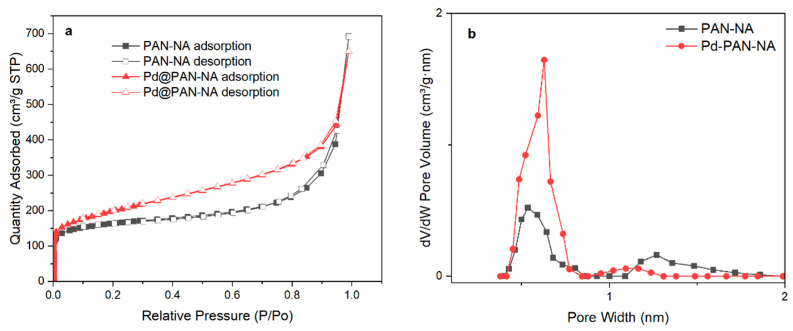
(**a**) N_2_ adsorption–desorption isotherms, and (**b**) porosity distribution via the original density functional theory of PAN-NA and Pd@PAN-NA.

The elemental composition of Pd@PAN-NA was studied using EDX analyses. As presented in Figure 3, the Pd@PAN-NA catalyst is composed of C, N, O, and Pd elements. Also, the elemental mapping confirmed that the Pd nanoparticles are well dispersed in the polymer. The porous properties of the PAN-NA support were studied after the loading of Pd NPs. As can be seen in Figure 4a, according to IUPAC classifications, the N_2_ uptakes in the supported polyaminal and Pd@PAN-NA were type I isotherms. There were no hysteresis loops observed, indicating that polymers have reversible adsorption–desorption isotherms. The N_2_ adsorption isotherm does not change after the introduction of the Pd nanoparticles, which means that the Pd NPs do not break the porous skeleton of the PAN-NA support. The total pores volume of the Pd@PAN-NA catalyst (1 cm^3^/g) was reduced by only 0.07 from the original value. As the size of Pd nanoparticles is much larger than the pores diameters of the PAN-NA support, the external pores may be blocked by the Pd NPs, which is responsible for the increscent in the BET surface area (607/707 m^2^/g, PAN-NA/Pd@PAN-NA). The pores size of the Pd@PAN-NA catalyst is centered at 0.62 with a narrow distribution (Figure 4b). Nevertheless, the distribution of pore size has not altered a lot after the loading of Pd NPs. These findings indicate that the microporosity of the Pd@PAN-NA catalyst is preserved, and thus, it is suitable for the catalytic application.

### 3.2. Methylene Blue Reduction

**Figure 5:**
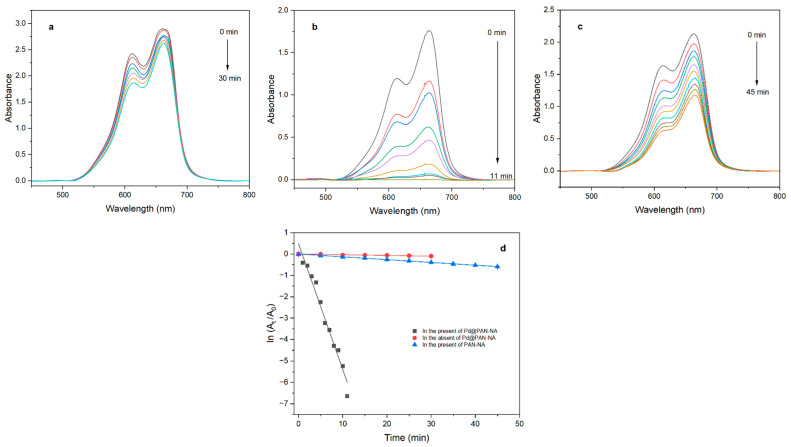
UV-vis spectra of MB degradation (Variation of the full MB spectrum at various times) (**a**) in the presence of Pd@PAN-NA, (**b**) in the absence of Pd@PAN-NA, and (**c**) in the presence of PAN-NA only. (**d**) The plot of the pseudo-1st-order model for MB degradation in the three cases (**a**–**c**).

The catalytic activity of the Pd@PAN-NA catalyst was evaluated toward methylene blue as a carcinogenic organic contaminant via the reduction mechanism [36]. Firstly, the reduction in MB with NaBH_4_ was examined in the absence and presence of the nano catalysts. MB shows a strong characteristic absorption peak at λ_max_ = 664 nm resulting from n-π* transition, and a peak at λ_max_ = 614 nm resulting from π-π* transition [37]. In the absence of the Pd@PAN-NA catalyst (Figure 5a), the characteristic peak reduces slowly and the degradation of methylene blue by NaBH_4_ takes a very long time (only 9.5% of the dye has been degraded after 30 min) due to the thermodynamically favorable process [38]. In the existence of Pd@PAN-NA (0.05 mL, 2 mg/mL), rabid degradation occurred, and the blue color of MB turned colorless (leuco MB). The MB was completely reduced (96%) within 11 min (Figure 5b).

To further confirm the role of Pd nanoparticles for the effective reduction in MB, the supported polymer (PAN-NA) was tested in the presence of NaBH_4_ (Figure 5c). Upon using a high concentration of the polymer (0.05 mL, 5 mg/mL), the degradation of MB takes place at a slow rate, and 84.8% of the dye was degraded after 45 min. This observation proves the promising catalytic performance of Pd nanoparticles. In addition, the synergy between the two materials (nanoparticles and support) led to an improvement in the chemical reduction efficiency of the Pd@PAN-NA catalyst.

#### 3.2.1. Kinetic Study

The rate of the reduction reaction is dependent on the MB concentration and independent from the sodium borohydride, as NaBH_4_ exists in the solution with higher amounts as compared to MB. Thus, the reduction reaction follows a pseudo-1st-order kinetic model [39,40]. The linear equation is expressed as follows:

$$\ln\left(\;\frac{A_{t}}{A_{0}}\;\right)=-k_{\mathrm{app}}\;t$$

where *k*_app_ is the apparent rate constant (min^−1^) and t is the reduction time (min). Figure 5d represents the pseudo-1st-order fitting data of the MB reduction in the absence and existence of the Pd@PAN-NA catalyst and the existence of only the PAN-NA support. By plotting the t vs. ln(A_t_/A_0_), the apparent rate constant was calculated from the slope. The *k*_app_ value in the case of the existing Pd@PAN-NA catalyst was (0.588 min^−1^), which is higher by 179.8 times than that in the absence of Pd@PAN-NA (0.003 min^−1^) and 44.8 times than that of the existing PAN-NA polymer (0.013 min^−1^). These results illustrate the impact of the Pd nanoparticles which are immobilized onto the polymer in speeding up the degradation rate of the MB dye.

#### 3.2.2. Effect of Catalyst Dose

**Figure 6:**
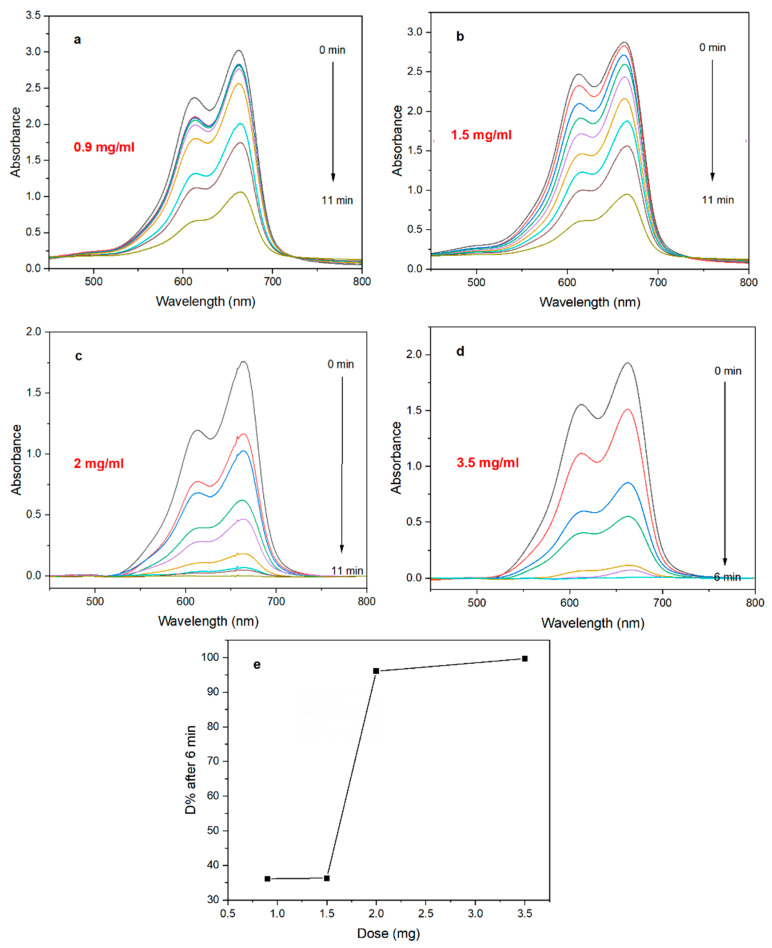
(Variation of the full MB spectrum at various times) at different catalyst doses (**a**–**d**). (**e**) The effect of the catalyst dose on the MB degradation efficiency.

To study the influence of the catalyst dose on increasing the reduction rate of MB, the experiments were performed under the following reaction conditions: methylene blue concentration of 0.1 mM, NaBH4 mass of 3 mg, and catalyst amount of 0.05 mL with different concentrations (mg/mL) of 0.9, 1.5, 2 (initial), and 3.5, respectively. The MB chemical reduction was monitored using a UV-vis spectrophotometer and the data were plotted as wavelength (nm) vs. absorbance, as shown in Figure 6a–e.

Generally, increasing the concentration of the catalyst results in increasing the available active site being ready for the adsorption of the reactants. Therefore, the chemical reduction takes place in a short reaction time [41,42,43]. As can be seen in the figure below, upon the increase in the catalyst dose from (0.9–3.5 mg/mL), the degradation efficiency of methylene blue increased from 36.1% to reach a maximum degradation value of 99.6% after a reaction time of 6 min and a catalyst dose of 3.5 mg/mL. Thus, the catalyst dose of 3.5 mg/mL was the most effective in the MB reduction and it was selected for the next experiments.

#### 3.2.3. Effect of pH

**Figure 7:**
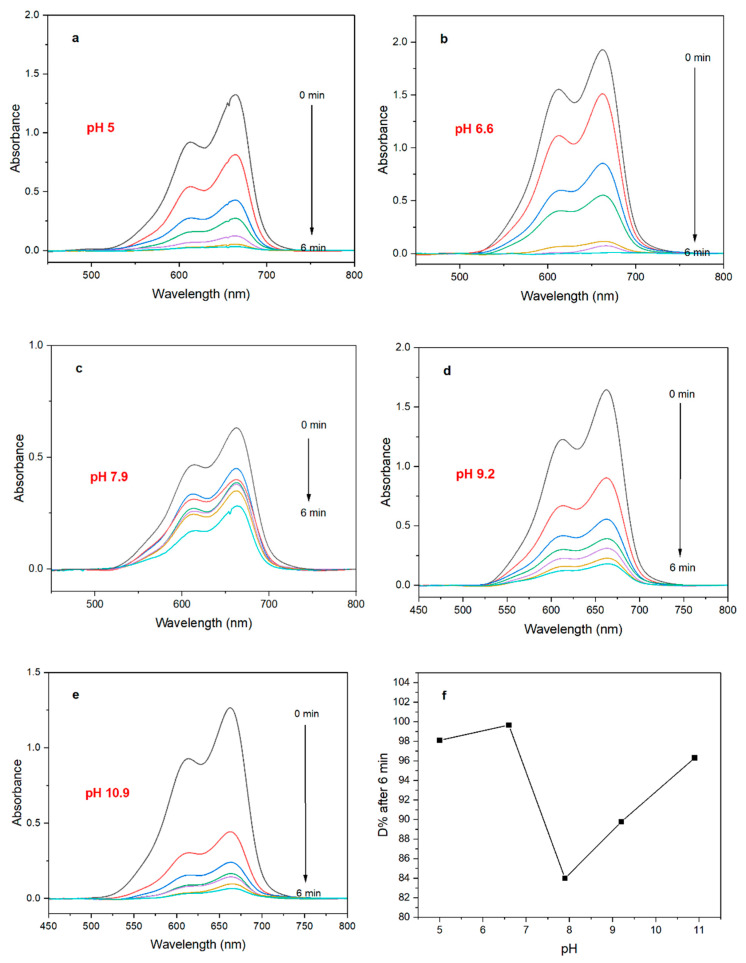
(Variation of the full MB spectrum at various times) at different pH values (**a**–**e**). (**f**) The effect of pH on the MB degradation efficiency.

The medium pH is another significant factor that affects the chemical reduction in MB. The pH values of 5, 6.6 (initial), 7.9, 9.2, and 10.9 was adjusted using 0.1 M of the NaOH and HCl solutions, and the other parameters were kept unchanged (initial dye concentration of 0.1 mM, NaBH_4_ mass of 3 mg, catalyst dose of 3.5 mg/mL, and time of 6 min). Figure 7a–e represents the UV-vis spectra of the MB degradation at each pH value. The higher degradation efficiency was found in the low acidic and neutral medium, specifically at the pH of 5 and 6.6 with D% values of 98.1% and 99.6%, respectively (Figure 7f). It should be mentioned that the pH lower than 5 is not included in this test because NaBH_4_ decomposes rapidly at a very low pH medium [15]. A pH of 6.6 was fixed for the following experiments.

#### 3.2.4. Effect of Dye Concentration

**Figure 8:**
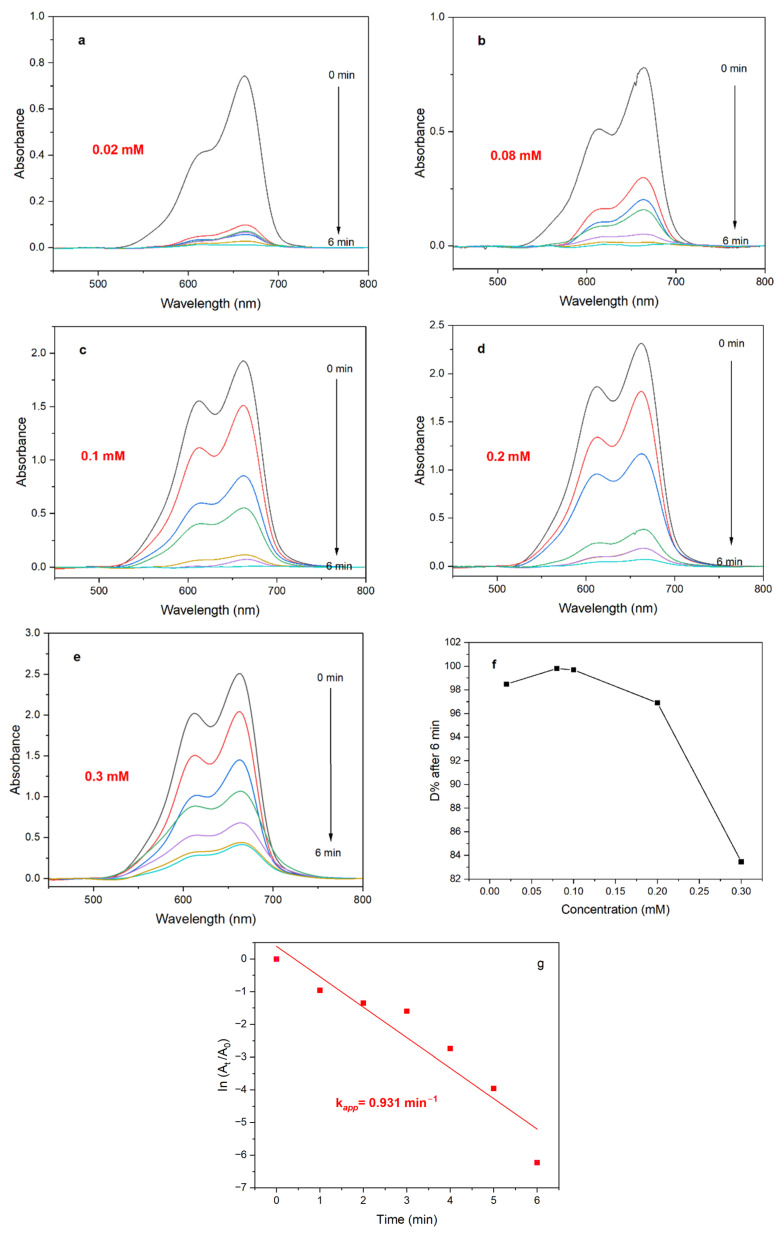
(Variation of the full MB spectrum at various times) at different dye concentrations (**a**–**e**). (**f**) The effect of the dye concentration on the MB degradation efficiency. (**g**) The plot of the pseudo-1st-order model for MB degradation at the optimum conditions.

To evaluate the influence of the initial dye concentration on the catalytic reduction in MB, different solutions of MB were prepared with concentrations (mM) of 0.02, 0.08, 0.1 (initial), 0.2, and 0.3. As can be seen in Figure 8a–e, the decrease in absorbance of each MB solution was recorded within a 6 min reaction time. The MB degradation efficiency was first raised as the dye concentration increased in the solution. After the D% reaches a maximum, any further increase in the dye concentration reduces the degradation efficiency (Figure 8f). This behavior can be explained as follows: at low dye concentrations, the large number of available active sites are ready to adsorb a large number of dye molecules. Thus, upon increasing the MB concentration, the reduction rate increases as well. At the maximum D%, the catalyst active sites are completely saturated with the dye molecules and there are no additional sites available for the unabsorbed molecules that come with the increase in the dye concentration. Therefore, the reduction reaction takes a long time, which contributes to decreasing the degradation efficiency. Moreover, the competition between the dye molecules and the reaction products increases with the increasing concentration, which makes it difficult for the reactants to reach the reaction sites, which in turn leads to a decrease in the catalytic activity [44]. The highest D% values of 99.8% and 99.6% were noticed at 0.08 and 0.1 mM of the dye concentration, respectively. After reaching the optimum reaction conditions for the chemical reduction in MB, the *k*_app_ was calculated as a pseudo-1st-order kinetic model plot at 0.08 mM (Figure 8g). Additionally, the *k*_app_ value was compared with previous studies, as shown in Table 1.

#### 3.2.5. Mechanism of Methylene Blue Reduction

The chemical reduction in MB from a blue color (oxidized form) to colorless (reduced form) is postulated to occur via the following mechanism: both reactants (MB and BH_4_^−^) diffuse through the pores of the polymer support and interact with the surface of the Pd nanoparticles. The BH_4_^−^ ions release electrons to the Pd surface and produce protons. The Pd nanoparticles receive the electrons and transfer them to the adsorbed MB. The dye molecules get the electrons and protons and are reduced to form colorless LMB. Then, the reaction products are desorbed from the catalyst surface to the aqueous solution [37,51,52].

## 4. Conclusions

In summary, the Pd nanoparticles were immobilized into the polyaminal-based porous organic polymer via a reversible double solvent approach. The catalyst showed good dispersity and enhanced thermal stability, as illustrated by using the thermogravimetric and EDX methods. Pd@PAN-NA as an efficient catalyst was used to reduce methylene blue dye from wastewater. Upon using a dye concentration of 0.08 mM, a catalyst dose of 3.5 mg/mL, and a pH of 6.6 reaction conditions, the catalyst degraded 99.8% of the dye within only 6 min. These results demonstrate the superior catalytic properties of the Pd@PAN-NA catalyst in the removal of organic dyes from waste effluent.

## Figures and Tables

**Table 1:** Comparison of the catalytic activities of Pd@PAN-NA toward MB with previous studies.

Entry	Catalyst	Conditions	Time (min)	*k*_app_ (min^−1^)	Ref.
1	Pd@PAN-NA	[MB] = 0.08 mM [Catalyst] = 3.5 mg/mL	6	0.931	This work
2	Fe/CC-CH	[MB] = 0.05 mM [Catalyst] = 5 wt%	9	0.280	[45]
3	MTiCuPd500	[MB] = 2 g/L [Catalyst] = 5 mg	20	-	[46]
4	AgNPs	[MB] = 0.001 M [Catalyst] = 0.01 M	13	-	[47]
5	Au@TiO_2_	[MB] = 0.04 mM [Catalyst] = 2 mg	12	0.156	[48]
6	CuO	[MB] = 1 mM [Catalyst] = 1 mg	12	0.419	[49]
7	rGO-SiW	[MB] = 35 mg/L [Catalyst] = 0.1 mg/mL	34	0.055	[50]

## Data Availability

No data were used to support this study.
